# Pathogenic Mechanisms of *Fusobacterium nucleatum* on Oral Epithelial Cells

**DOI:** 10.3389/froh.2022.831607

**Published:** 2022-04-05

**Authors:** Sabine Groeger, Yuxi Zhou, Sabine Ruf, Joerg Meyle

**Affiliations:** ^1^Department of Periodontology, Justus-Liebig-University of Giessen, Giessen, Germany; ^2^Department of Orthodontics, Justus-Liebig-University of Giessen, Giessen, Germany

**Keywords:** *F. nucleatum*, oral epithelial cells, immune response, cytokines, receptors, infection, cancer, periodontitis

## Abstract

Periodontitis is an oral chronic inflammatory disease and may cause tooth loss in adults. Oral epithelial cells provide a barrier for bacteria and participate in the immune response. *Fusobacterium nucleatum* (*F. nucleatum*) is one of the common inhabitants of the oral cavity and has been identified as a potential etiologic bacterial agent of oral diseases, such as periodontitis and oral carcinomas. *F. nucleatum* has been shown to be of importance in the development of diverse human cancers. In the dental biofilm, it exhibits a structural role as a bridging organism, connecting primary colonizers to the largely anaerobic secondary colonizers. It expresses adhesins and is able to induce host cell responses, including the upregulation of defensins and the release of chemokines and interleukins. Like other microorganisms, its detection is achieved through germline-encoded pattern-recognition receptors (PRRs) and pathogen-associated molecular patterns (PAMPs). By identification of the pathogenic mechanisms of *F. nucleatum* it will be possible to develop effective methods for the diagnosis, prevention, and treatment of diseases in which a *F. nucleatum* infection is involved. This review summarizes the recent progress in research targeting *F. nucleatum* and its impact on oral epithelial cells.

## Introduction

The oral epithelium as part of the masticatory mucosa, the lining mucosa, and the specialized mucosa, provides a barrier that separates the oral soft tissues from the environment. This barrier is the result of a number of functional and structural protein interactions that result in the capability to react to numerous exogenous, possibly toxic, influences [1]. It is actually known that epithelial cells are not only passive bystanders, but are able of responding to external stimuli by producing a number of cytokines, adhesion molecules, growth factors, chemokines, and matrix metalloproteases [2]. *Fusobacterium nucleatum*, a Gram-negative obligate anaerobic bacterium, belongs to the genus *Fusobacterium*, which normally lives parasitically in the oral cavity, urogenital tract, soil, intestinal tract, and upper digestive tract, but most commonly in oral plaque. It is frequently present in the oral cavity, in diseased as well as healthy individuals [3].

In the past, *F. nucleatum* has been regarded as a component of the normal flora of the human body. Due to the continuous isolation of the bacterium from clinical samples, *F. nucleatum* has attracted attention from researchers and has been recognized as a bacterium that should not be ignored. It was verified that *F. nucleatum* is an opportunistic pathogen with strong pathogenicity. *F. nucleatum* is frequently detected in oral and systemic infections. It is associated with various human diseases, such as periodontitis, angina, lung abscesses, chronic otitis, sinusitis, peritonsillar abscesses, cerebral abscesses, inflammatory bowel disease, ulcerative colitis, Crohn's disease, gynecological abscesses, neonatal sepsis, Lemierre's syndrome, and infective endocarditis [4–9].

In oral infections, it is widely present in infected dental pulp, periodontal, and other inflammatory lesions. Most importantly, *F. nucleatum* is implicated in different kinds of periodontal disease from the reversible and rather mild form of gingivitis to the more severe forms of periodontitis such as chronic and aggressive periodontitis [3]. The prevalence of F. nucleatum is higher in severe forms of the disease, with progressive inflammatory responses, and increased pocket depth. [10].

**Figure 1:**
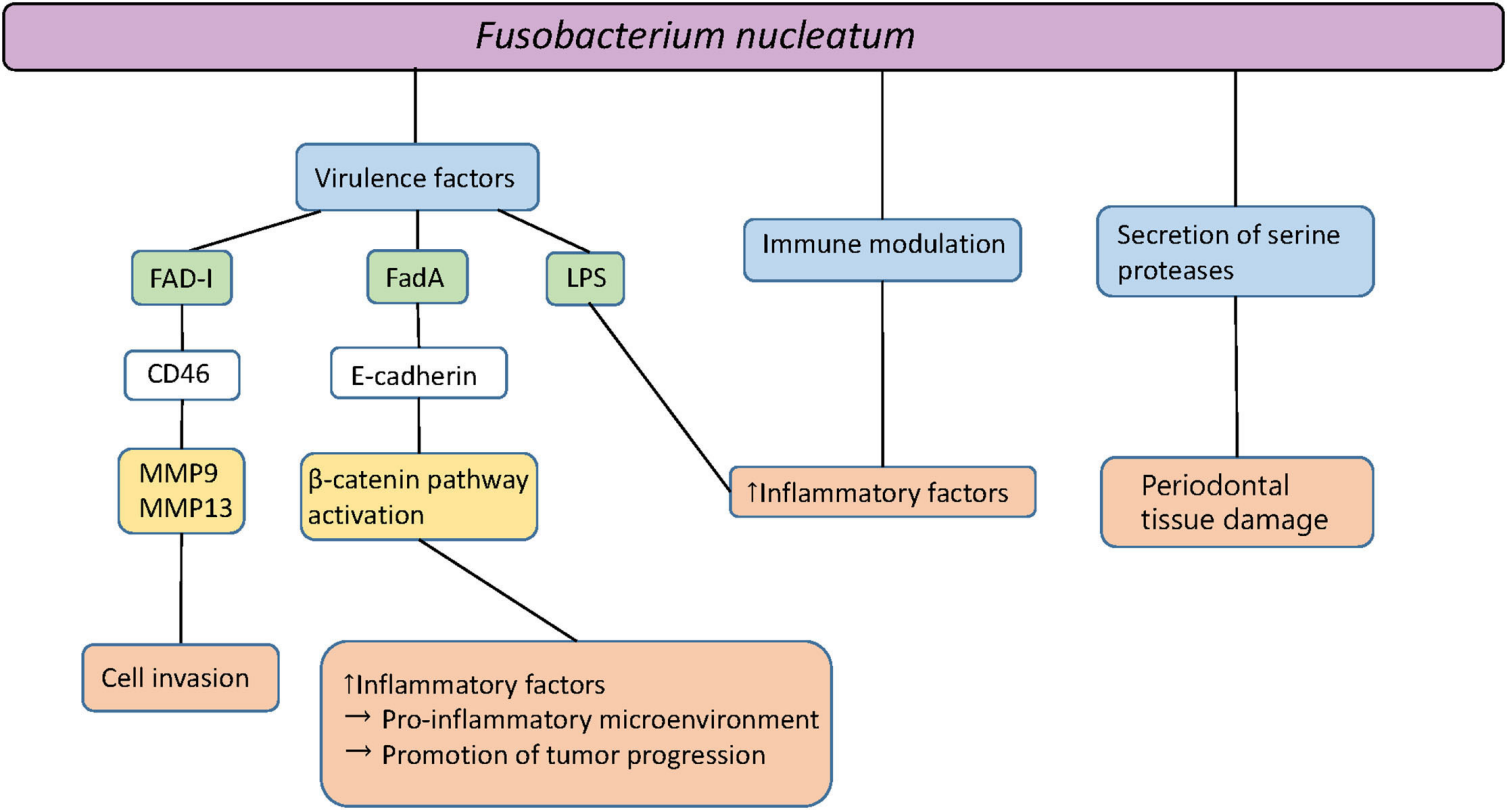
Pathogenicity of *Fusobacterium nucleatum*. Fad-I, *Fusobacterium*-associated defensing inducer; FadA, Fusobacterial adhesion; MMP, Matrix metalloproteinase; LPS, Lipopolysaccharides.

*F. nucleatum* is not only detected in periodontal sites but additionally in saliva, with higher prevalence in patients exhibiting gingivitis and periodontitis, in comparison to healthy controls [11]. The results of animal studies suggest a causative role of *F. nucleatum* in periodontal infections [3]. A mono-infection of mice with *F. nucleatum* induces periodontal bone loss or abscess formation [12]. Together with other oral species, synergistic effects of virulence are detected, resulting in increased bone loss, abscess development, or death [13]. It has been shown that *F. nucleatum* passes through the gingival epithelial cells to the endocytic pathway of degradation after the invasion. After this event, no cytopathic effect on gingival epithelial cells persists, which may be associated with the host evasion strategies of *F. nucleatum* in the pathogenesis of periodontitis [14]. This oral gram-negative bacterium can enter connective tissue and enhance tissue destruction through proteases as well as by inducing abundant inflammatory responses [14]. Figure 1 shows an overview of the multiple forms and components of *F. nucleatum* and their interactions with oral epithelial cells.

## *F. nucleatum*—A Bridging Organism

In the dental bacterial biofilm, F. nucleatum plays a structural role by acting as a bridging organism, connecting the primary colonizers such as the *Streptococcus* species to the mostly anaerobic secondary colonizers to which it can bind as well, i.e., *Porphyromonas gingivalis* (*P. gingivalis*) and *Aggregatibacter actinomycetemcomitans* (*A. actinomycetemcomitans*) [3, 15, 16]. *F. nucleatum* exists in the intermediate layer of tooth-attached human plaque samples, as proposed by Kolenbrander and London [17], which also was supported by an *in vivo* study [18]. It can co-aggregate with almost all bacterial species that participate in oral plaque formation [19]. *F. nucleatum* is able to bind to and transport *Streptococcus cristatus*, a non-invasive bacterial species, into host cells, working as a shuttle [20]. In general, known fusobacterial adhesins (Aid1, CmpA, Fap2, FomA, FadA, and RadD) play a vital role in microbial coaggregation, mediating the invasion and facilitating the spread of bacteria [3, 6, 21–24].

RadD plays an eclectic role in fusobacterial adhesion, which has been shown as the main adhesin that mediates the attachment of numerous gram-positive early colonizers [23] and promotes fusobacterial adherence to biofilms [25]. It can bind to the *S. mutans* adhesin SpaP to mediate the co-aggregation of these two bacterial species and progressive biofilm organization [15, 26]. It conveys interactions not only to bacterial species but also to *Candida albicans*, which can also be part of the oral microbiota [15, 23, 27]. According to a microarray analysis, Kaplan et al. found that a small hypothetical protein encoded by FN1253, which they designated as Adhesion Inducing Determinant 1 (Aid1), is induced in *F. nucleatum* single-species biofilms. Aid1 appears to be unique to fusobacteria and potentially plays a vital role in facilitating RadD-mediated interaction with oral streptococci [28]. The interaction between *F. nucleatum* ATCC 23726 and *S. gordonii* V288 is mediated by RadD and another second outer membrane protein called Coaggregation-mediated Protein A (CmpA) [29]. While CmpA increased the expression under biofilm conditions, RadD expression was decreased, indicating that these two proteins may be involved in different physiological processes [29].

The fusobacterial adhesin (FadA) is the only adhesin that has been demonstrated to bind to host cells and, until now, remains to be the virulence factor from *F. nucleatum* that has been characterized the best [30]. FadA plays an essential role in inducing the tumorigenic responses and binding and invasion of host cells by the organism [31]. A synthetic peptide that can prevent FadA from binding to E-cadherin inhibits tumorigenic responses in colorectal cancer [32]. FadA binds to E-cadherin, activates β-catenin signaling, and regulates the inflammatory and oncogenic reactions in a differential manner [32–35]. *F. nucleatum* activates p38 MAP kinase followed by the secretion of matrix metalloproteinase (MMP)−9 and MMP-13 [33, 36–38]. MMP-9 and MMP-13 induce invasion and metastasis manifestation [6, 39]. This supports the migration of cells over the stimulation of Etk/BMX, S6 kinase p70, and RhoA kinase [40]. Liu et al. assessed the prevalence of *F. nucleatum* and its virulence factor FadA adhesion gene (*fadA*) in subgingival biofilm samples from patients with gingivitis or periodontitis with or without fixed orthodontic appliances. It was found that *F. nucleatum fadA* was detected in higher amounts in the periodontitis group and non-orthodontic gingivitis group compared to the other groups. It was also increased in the orthodontic gingivitis group but only compared to healthy subjects. The authors concluded that *F. nucleatum* carrying *fadA* probably plays a role in the development of non-orthodontic gingivitis and periodontitis compared with orthodontic gingivitis [41].

FomA is a major outer membrane pore protein of *F. nucleatum*. The increased abundance of FomA may be related to the promotion of biofilm formation [42]. FomA was also identified as the target antigen bound to *F. nucleatum*-specific IgA [43]. The production of anti-FomA IgG antibodies was dependent on TLR2 expression [44]. F. nucleatum invades host cells through a “zipper” mechanism that relies on a large number of adhesins [45]. However, a study found that *P. gingivalis* outer membrane vesicles (OMVs), reduced the expression levels of FadA and FomA through protease components and further inhibited the invasion of *F. nucleatum* into the oral epithelial cells [46].

Fibroblast activation protein 2 (Fap2) is a galactose-sensitive hemagglutinin and adhesin that probably participates in the virulence of Fusobacteriae [22]. Its function is critical in mediating colorectal cancer (CRC) development through binding with acetylgalactosamine (Gal-GalNAc), which is overexpressed in human metastases and colorectal adenocarcinoma [47]. It was demonstrated that the Fap2 protein of *F. nucleatum* directly interacts with the T cell immune receptor with Immunoglobulin G (TIGIT) expressed on NK cells and tumor-infiltrating lymphocytes, causing inhibition of natural killer (NK) cell cytotoxicity and lymphocytes activity, thus inducing the development of CRC [48, 49]. FadA interacts with endothelial cells and epithelial cells, while Fap-2 binds only to Gal-GalNAc [6, 50].

Compared with *P. gingivalis, F. nucleatum* demonstrated a highly invasive capacity [14]. The coinfection with *F. nucleatum* can enhance the adhesion and invasion of *P. gingivalis* and *A. actinomycetemcomitans* to human gingival epithelial cells and inhibits host innate immune responses [51]. The green and black tea extracts, epigallocatechin-3-gallate (EGCC), and theaflavins diminish the adherence of *F. nucleatum* to oral epithelial cells and matrix proteins [52]. Furthermore, these tea components also inhibit *F. nucleatum*-mediated hemolysis and hydrogen sulfide production, which represent two further virulence factors expressed by this bacterium [52].

## *F. nucleatum*—Receptor Interactions

Microbial detection is achieved through germline-encoded pattern-recognition receptors (PRRs) and pathogen-associated molecular patterns (PAMPs) that surveil the extracellular as well as the intracellular area for conserved microbial components that indicate infection [53–55]. Representative bacterial PAMPs include lipid A of lipopolysaccharides (LPS), lipopeptide, and peptidoglycans (PGNs) [54]. The classification of the most PRRs is based on protein domain homology, discriminating them into one of five families, including Toll-like receptors (TLRs) and nucleotide-binding domain, leucine-rich repeat (LRR)-containing [or nucleotide-binding oligomerization domain-containing proteins (NOD)-like] receptors (NLRs) [55, 56]. TLRs are membrane-bound inborn receptors, while NLRs form the unbound intracellular receptor class [55].

Toll-like receptors are an essential class of protein molecules that participate in innate immunity, and they are also the bridge connecting innate and adaptive immune responses. TLR 1, 2, 4, 5, 6, and 10 are expressed on the cell surface and are transferred to phagosomes after being activated, whereas TLR 3, 7, 8, and 9 are expressed in intracellular compartments in nearly all cell types, principally in the endosomes and the endoplasmic reticulum, with the ligand-binding domains sampling the lumen of the vesicle [57]. In the oral cavity, both immune cells and cells of the periodontium express TLRs [58]. Gingival keratinocytes express TLR2 [59], which interacts with either TLR1 or TLR6 [54]. TLR2 recognizes gram-positive and gram-negative bacterial PAMPs and activates intracellular signaling pathways, which could then induce antimicrobial peptides, like human beta defensins (hBDs) and inflammatory markers like cytokines or MMPs [54, 59, 60]. TLRs on the epithelial cell surface recognize *F. nucleatum* which activates pro-inflammatory signaling pathways [33]. The hBD2 and hBD3 are induced in oral epithelial cells *via* TLR2 and neutrophilic alkaline phosphatase (NALP) 2 [14, 53, 61]. Ji et al. demonstrated that TLR2 and NALP2 mediate the induction of hBDs, but not IL-8, and hBD2 and hBD3 are divergently regulated [53]. *Fusobacterium*-associated defensin inducer (FAD-I) is the principal *F. nucleatum* agent for hBD2 induction in human oral epithelial cells *via* TLR-1/2 and TLR-2/6 [31].

NOD-like receptors are normally composed of a central conserved NOD domain, a C-terminal LRR, and an N-terminal effector domain [62, 63]. Depending on the N-terminal effector domain, NLRs can be categorized into varying groups, such as NLRP, NLRC, and NLRX [64]. Every NLR is essential and not redundant in recognition of specific PAMPs or damage-associated molecular patterns (DAMPs). The responses that are induced can be classified as inflammasome-dependent or -independent [62]. NLRP1, NLRP3, and NLRC4 for example can build inflammasomes to activate caspase-1 after the sensing of PAMPs and DAMPs that results in the release of IL-1β [62, 65]. NLRP10 is the smallest human nucleotide-binding and leucine-rich repeat (NLR) protein which has anti- and pro-inflammatory functions [66]. It takes part in activating the extracellular signal-regulated kinases (ERK) signaling pathway in human epithelial cells infected with *F. nucleatum* and augments the pro-inflammatory cytokine IL-1α levels [66]. NOD proteins are cytosolic pattern recognition molecules that belong to the family of NLRs, recognizing PGN, a component of bacterial cell walls [67]. NOD1, NOD2, and NLRX1 induce pro-inflammatory responses through nuclear factor kappa B (NF-κB) or mitogen-activated protein kinase (MAPK) signaling during microbial infection [56, 64]. NOD1 is mostly involved in sensing components of Gram-negative bacteria, while NOD2 is able to recognize both Gram-negative and Gram-positive bacteria [68]. Both NOD1 and NOD2 might have played a role in recognizing periodontal pathogens, but the stimulatory activities of *P. gingivalis* are weaker than those of other periodontal pathogens [69]. In addition, the NLR family acts as an essential regulator of inflammatory and innate immune response, which can control IL-1, NF-κB, and host response to pathogens including distinct forms of cell death [70]. Depletion of NLRX1 can decrease *F. nucleatum* infection-activated NLRP3 in gingival epithelial cells (GECs). It is proposed that NLRX1 should enhance the innate immune response during infection by pathogens but behave as a break to prevent excessive inflammation under normal circumstances [62]. A study also found that *F.nucleatum* can stimulate NLRP3, activate upstream signal molecules of ATR-CHK1, and inhibit the activation of CHK1, promoting tumor growth and proliferation in squamous cell carcinomas (SCCs) [71].

Previous studies show that *F. nucleatum* can inhibit proliferation, apoptosis, reactive oxygen species (ROS), and inflammatory cytokine production partly of human gingival fibroblasts by activating the AKT/MAPK and NF-κB signaling pathways [72]. It is also reported that a large number of different genes are enriched in the PI3K/AKT signaling pathway after stimulation with *F. nucleatum* [72]. The activation of the PI3K/AKT pathway may induce cell growth [73, 74]. NF-κB, as the downstream molecule of the PI3K/AKT signaling pathway, acts synergetically in regulating cell proliferation, apoptosis, ROS generation, and the inflammatory respose [72], and it also mediates oral infections and periodontitis [75].

A recent study showed that *F. nucleatum* aggravated colitis by induction of the Th1-related cytokine IFN-γ over the AKT2 signaling pathway *in vitro* and *in vivo*. The group demonstrated that *F. nucleatum* could support the progression of colitis by proinflammatory M1 macrophage twisting. Therefore, *F. nucleatum* or AKT2 signaling may be therapeutic targets in order to inhibit the development of the disease [76].

**Figure 2:**
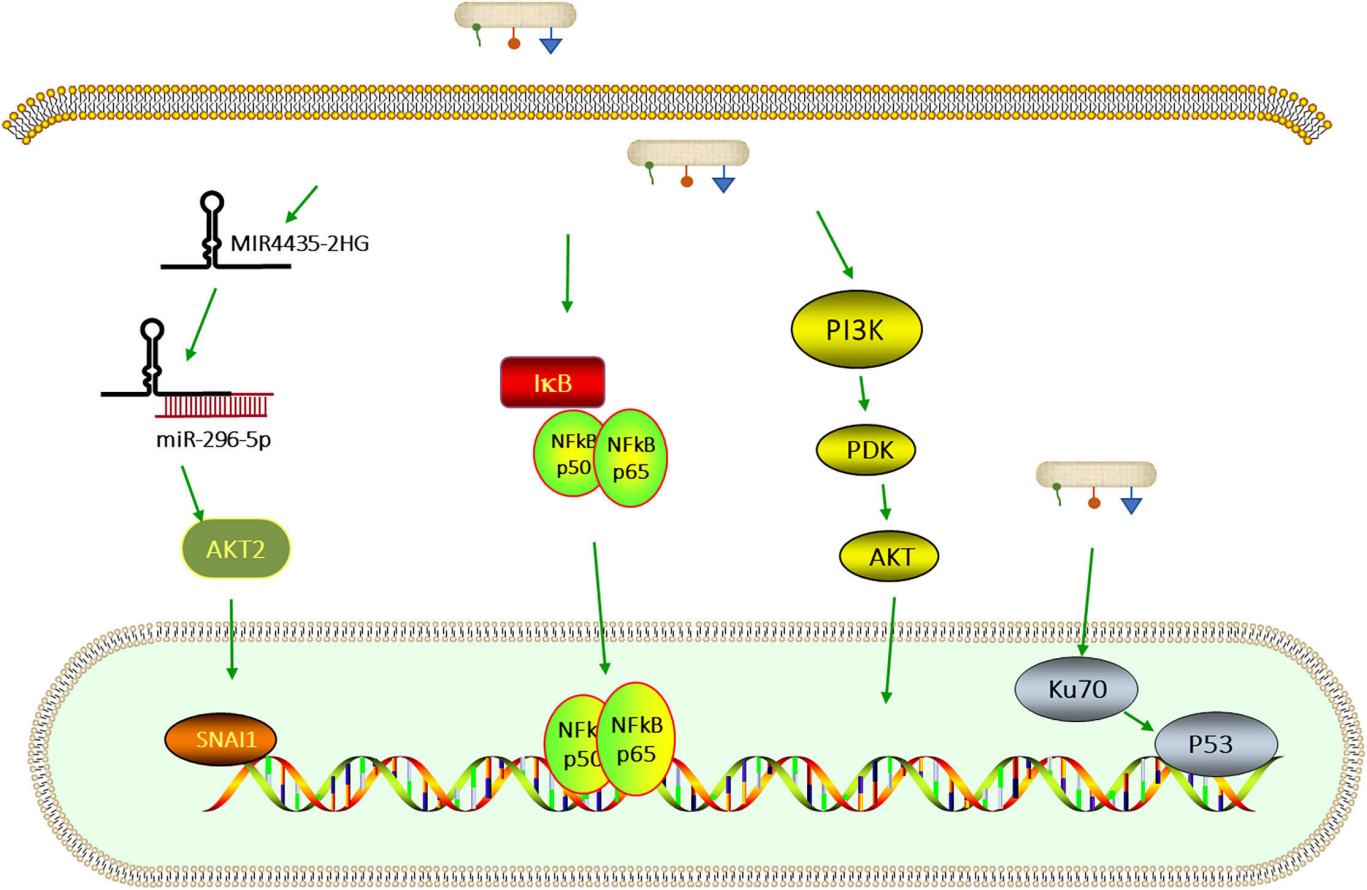
Signaling pathways that are related to *F. nucleatum* infection. *F. nucleatum* upregulates MIR4435-2HG, which binds miR-296-5p and weakens the inhibitory effect of miR-296-5p on SNAI1 *via* AKT2 [77]. *F. nucleatum* can also activate the PI3K/AKT—nuclear factor kappa B (NF-κB) signaling pathway which regulates cell proliferation, apoptosis, and the inflammatory response [72]. The infection with *F. nucleatum* promotes the capability of proliferation by leading to DNA damage through the Ku70/p53 pathway [78].

Some signaling pathways that are related to *F. nucleatum* are shown in Figure 2.

## *F. nucleatum* and Defensins

The barrier function of epithelial results from the unique structural integrity and the production of antimicrobial peptides, such as hBDs and a cathelicidin, LL-37 [79]. Human beta defensins are a family of epithelial cell-derived antimicrobial peptides which are of importance in immune defense against challenging pathogens. The epithelia of many body sites express hBD1 constitutively but express hBD2 and hBD3 under conditions of infection or inflammation [53, 80]. The influences of oral bacteria on the expression of hBDs in epithelial cells and the signaling pathway have been studied extensively. hBD2 was found to have strong bactericidal effects against gram-negative periodontal bacteria [81], while hBD3 also demonstrated activity against Gram-positive as well as Gram-negative bacteria [82]. Moreover, hBD2 and hBD3 can furthermore attract different immune cells and link together the innate and adaptive immune responses [83]. However, gingival epithelium can also express hBD2 in the absence of inflammation, presumably due to the constant exposure to oral bacteria [53]. The promoter region of hBD2 contains numerous regulatory elements, including the binding sites for NF-κB, activator protein (AP)-1, AP-2, and nuclear factor for IL-6 expression (NF-IL-6), whereas the promoter of hBD3 contains no discernible NF-κB binding elements [53, 84]. *F. nucleatum* and *F. nucleatum* cell wall (FnCW) extracts induce expression of hBD2 and hBD3 in cultured primary human GECs *in vitro* [85, 86]. The induction of hBD2 in GECs in response to FnCW occurs mainly through MAPK signaling, not NF-κB [87]. Krisanaprakornkit et al. demonstrated that hBD2 and Interleukin-8 (IL)-8 could also be induced by LPS extracted from the cell wall of *F. nucleatum* [85]. Another study supported that *F. nucleatum* infection induced the expression of hBD2 and hBD3 in gingival cells [86].

LL-37, the only cathelicidin-derived antimicrobial peptide found in humans [88], is expressed by neutrophils and epithelial cells, and in the gingiva, it is localized in the junctional epithelium [89]. The local deficiency of LL-37 in the gingival crevicular fluid is suggested to be a supporting factor in the pathogenesis of severe cases of periodontitis [90]. Direct killing of microbes by antimicrobial peptides, including LL-37, is thought to serve as a crucial innate immune defense mechanism to prevent the growth of microbes in the gingival sulcus [91]. In addition to its antimicrobial effects, LL-37 suppresses inflammatory responses and modulates the apoptotic behavior of neutrophils [91]. Studies found that LL-37 is able to suppress the effect of *P. gingivalis*-induced proinflammatory responses of human gingival fibroblasts in a paracrine manner, suggesting that inflammatory responses to *P. gingivalis* in the gingival tissue are suppressed by LL-37 *in vivo* [91]. Moreover, LL-37 has the ability to suppress periodontopathogenic LPS-induced IL-8 production in both human periodontal ligament fibroblasts and gingival fibroblasts [92]. However, after stimulation with *F. nucleatum*, LL-37 is only weakly expressed in an organotypic dento-epithelial model, which is used to mimic the dento-gingival junction *in vitro* [93]. That is a coherent observation since LL-37 is mostly released by neutrophils and doesn't originate from epithelial cells [94].

## *F. nucleatum* and Immune Responses

The gingival epithelium is the tissue that is primarily challenged by plaque-associated bacteria [86]. Mucosal epithelia not only are a passive protective barrier but also can initiate immune responses over secretion of a number of cytokines and chemokines [79, 86]. *F. nucleatum* can induce significant changes in the expression of genes related to immune defense responses [95]. Here, we reviewed the inflammatory and immune responses due to *F. nucleatum*.

Gingival epithelial cells gene expression was investigated after the stimulation with a commensal FnCW preparation and hBD2 peptide. The results revealed significant changes in the expression levels of genes associated with immune and defense responses. The 20 most highly up-regulated genes included CC chemokine-ligand 20 (CCL20), calcium-binding protein S100A7, skin-derived antileukoprotease (SKALP), IL 1 family member 9 (IL1F9), IL-8, chemokine (C-X-C motif) ligand (CXCL) 5, complement factor 3 (C3), IL-32, serum amyloid A (SAA) 1, small proline-rich protein (SPRR) 2C, and CXCL1. Fourteen out of 20 are cytokines, components of the innate immune system or inflammatory markers, antimicrobials, or protease inhibitors. Two genes that were also strongly up-regulated (small proline-rich proteins, SPRR2B, and SPRR2C) are associated with structural issues of the epithelial barrier. The most obvious down-regulated genes included cell cycle regulatory genes cell division cycle (CDC) 20, S-phase kinase-associated protein (SKP) 2, proliferating cell nuclear antigen (PCNA), polymerase epsilon (POLE) 2), and ubiquitin-proteasome-associated genes [ubiquitin associated protein 2 like (UBAP2L), proteasome 26S subunit, non-ATPase 11(PSMD11)] [96]. Genes up-regulated by FnCW include encoding antimicrobial peptides and proteins. Up-regulated genes of defense responses included chemokines IL-8, CXCL1, CXCL3, CXCL5, and CXCL10, which attract neutrophils, monocytes, macrophages, and lymphocytes. Furthermore, colony-stimulating factor (CSF) 2, and CSF3 that stimulate neutrophil development were up-regulated [96].

Neutrophils are involved in inflammatory processes and release proteases that induce tissue damage. Numerous protease inhibitors are strongly up-regulated as a reaction to FnCW [96]. The targets of these inhibitors are proteases released by neutrophils. This promotes the control of tissue damage and represents a protective mechanism if commensal bacteria are present [96, 97]. In addition to providing protection against neutrophil proteases, these protease inhibitors may also protect against bacterial proteases released by pathogens, such as *P. gingivalis* [96]. Genes that reduce NF-κB function, a major transcription factor participating in inflammatory responses, are up-regulated in GECs after stimulation with FnCW [96].

In conclusion, *F. nucleatum* not only induces the antimicrobial peptide hBD2 but also affects immune responses over the induction of chemokines as well as the apparent impairment of NF-κB function. *F. nucleatum* supports the maintenance of an intact mucosal surface by enhancing the transcription of a number of protease inhibitors that, when translated, work as active inhibitors that impede tissue damage by proteases secreted from neutrophils, which constantly migrate into the oral cavity by passing through the gingival crevice.

CCL20 is a 70-amino-acid chemokine that attracts immature dendritic cells and T cells *via* the chemokine receptor CCR6. It plays a role in the specific differentiation of lymphocytes, such as developing Th17 and Treg cells that migrate into inflamed periodontal tissues [98, 99]. Ghosh et al. detected that primary oral epithelial cells release CCL20 as a reaction to *F. nucleatum* [100]. They also demonstrated *in vitro* that the inducible defensins hBD-2, hBD-3, tumor necrosis factor-α (TNF-α), and IL-1 β could induce the release of CCL20 by human oral epithelial cells. ERK 1/2 and p38 are required in this process [100]. Interestingly, Yin et al. provided a new aspect of the bacteria-specific innate immune responses by epigenetic regulation [101]. They demonstrated that DNA methyltransferase (DNMT) and histone deacetylase expression were impaired in GECs treated with the oral pathogens *P. gingivalis* and *F. nucleatum* [101]. Pretreatment with DNMT inhibitor 5'-azacytidine enhanced hBD2 and CCL20 expression in *F. nucleatum* infected GECs [101].

In human epithelial cells, infection with *F. nucleatum* induces the upregulation of 12 kinases involved in cell migration, proliferation, and cell survival signaling, as assessed by the Kinetworks immunoblotting system [40, 102]. IL-1, IL-6, and IL-8 are pro-inflammatory cytokines induced in immune and non-immune cells, like gingival keratinocytes [54]. *F. nucleatum* effectively stimulates inflammatory cytokines, IL-6, IL-8, and TNF-α [3, 36, 103, 104]. *F. nucleatum* infection in gingival epithelial cells can also activate NF-κB, which as consequence translocates to the nucleus, and there stimulates the expression of pro-inflammatory genes, such as genes encoding pro-IL-1β [39, 65]. Inflammatory cytokines such as IL-1β, IL-6, and TNF- α mainly cause periodontal tissue damage. IL-1β may also be involved in bone resorption and attachment loss which are characteristic properties of periodontitis.

Lipopolysaccharides from *F. nucleatum* is responsible for activating the immune system at the cellular level in periodontitis [33]. It can drive the production of inflammatory cytokines, such as IL-1α, IL-1β, IL-6, IL-8, and MMPs through the activation of the translocation of the NF-κB gene into the nucleus, activating immunological response and leading to the loss of periodontal attachment and tissue damage [6, 33, 104–108].

A recent study revealed that periodontitis enhanced gingival levels of IL-6 and CXCL2 in an animal model. Orthodontic tooth movement enhanced microbial-induced periodontal destruction and gingival IL-6 gene expression. Enhanced IL-6 and CXCL2 levels have also been detected in the gingiva in human periodontitis. Moreover, mechanical stress enhanced the stimulatory impact of *F. nucleatum* on IL-6 production *in vitro* [109].

*Fusobacterium nucleatum* infection can induce some DAMPs that mediate the formation of inflammasomes such as the high-mobility group box-1 protein (HMGB1) and apoptosis-associated speck-like protein (ASP) with a similar time-course as caspase-1 activation [6, 65, 110]. These data are consistent with animal studies in BALB/c mice infected with *F. nucleatum*, which suggested that infection with *F. nucleatum* is followed by fast induction of inflammation, the release of DAMPs, and macrophage infiltration in gingival tissues. The results also suggested that osteoclasts possibly drive bone resorption in the early stages of the inflammatory process [111]. HMGB1, a DNA-binding nuclear protein, is released actively after cytokine stimulation and passively during cell death; it is involved in several inflammatory disorders, cell adhesion, and cell migration [112]. HMGB1 can link with other molecules, including cytokines and TLR ligands. It activates cells by the differential engagement of numerous surface receptors such as TLR2, TLR4, and receptor of advanced glycation end-product (RAGE) [112]. RAGE is a receptor that binds structurally diverse molecules, and its signaling pathway includes the activation of MAPKs, NF-κB, PI3K/AKT, JAK/STAT [6]. The interaction between HMGB1 and RAGE may contribute to oral inflammation and oral cancer [6].

A disintegrin and metalloproteinase 8 (ADAM8), which is localized within the gingival epithelium, exhibits enhanced expression in inflamed tissues affected with chronic periodontitis [113]. ADAM8 mRNA expression in GECs is significantly induced by stimulation with *F. nucleatum* [113]. These findings suggest a possible role of ADAM8 in the innate immunity of periodontal tissues [113]. Moreover, ADAM8 is found significantly elevated in the gingival crevicular fluid of patients with chronic periodontitis [114] and synovia of patients with rheumatoid arthritis [115]. This is consistent with previous experiments which demonstrated that overexpression of ADAM8 may increase *in vitro* osteoclast development and function and cause bone resorption in mouse models [116, 117], implying a possible role of ADAM8 in the promotion of bone destruction [113].

## The Role of *F. nucleatum* in Oral Cancer

It is not a new idea that the microbiota plays a role in cancer. Bacteria, parasites, and viruses are all linked to the initiation and progression of cancer [15]. Chronic infections and persistent inflammation are linked to an augmented risk of cancer [1]. Persisting bacterial components may lead to up-regulation of immune-suppressing receptors, which can facilitate the ability of cancer cells to escape from anti-tumor responses of the host [1]. During the past decade, it was shown that oral bacteria might promote different oral diseases. The role of *F. nucleatum* as an inflammation-causing and cancer-inducing agent in oral epithelial cells is still emerging.

In a murine model of periodontitis-associated oral tumorigenesis that Binder Gallimidi et al. used, it was demonstrated that chronic bacterial infection promotes oral squamous carcinoma cells (OSCC) and intensified signaling over the IL-6-STAT3 axis [39]. *P. gingivalis* and *F. nucleatum* can induce tumorigenesis by directly interacting with oral epithelial cells *via* TLRs [39]. In contrast to *P. gingivalis*, which has been demonstrated to exhibit specific virulence factors that are probably involved in different stages of carcinogenesis, the presumed carcinogenic potential of *F. nucleatum* has been mostly explained over its pro-inflammatory effect by inducing different cytokines, including TNF-α, IL-6, IL-8, IL-10, IL-12, and production of ROS within the colon lining epithelial cells. This process ultimately can cause dysplasia and the development of cancer [3, 118]. *F.nucleatum* was also highly abundant in salivary samples and tumor sites of patients suffering from oral/head and neck squamous cell carcinoma [119, 120]. Tumor lesions showed a 6% higher abundance of *Fusobacterium* (95% CI, 3–9) than in non-tumor lesions and a 2.93 times higher chance of *Fusobacterium* being present in lesions [121]. These findings suggest that *Fusobacterium* infection might promote oral/head and neck cancer [121]. Recent studies demonstrated that *F. nucleatum* infection might impact cell migration, proliferation, invasion, and apoptosis, in/of gingival epithelial cells or OSCC [72, 78, 122].

In 2020, a cellular model of human immortalized oral epithelial cells (HIOECs) with *F. nucleatum* infection was established. The study group reported that *F. nucleatum* facilitated the functional loss of E-cadherin, cell migration, and the up-regulation of Snail family transcriptional repressor 1 (SNAI1) in non-cancerous as well as cancerous oral epithelial cells, which is regarded as an indicator for epithelial-mesenchymal transition (EMT) [77]. This model was used to perform high-throughput sequencing of cells infected by *F. nucleatum*. The function of the database of essential genes (DEGs) and cis targeted genes of differentially expressed long non-coding RNAs (lncRNAs) were shown to be significantly enriched in biological processes of DNA-templated transcription. In contrast, the trans-targeted genes of lncRNAs showed a main association with cell-cell adhesion and cell division. The tumor-associated genes were specifically analyzed and the top 10 hub genes related to tumor progression were identified. Some of the hub genes were also demonstrated to be aberrantly expressed in clinical samples of OSCC [123]. *F. nucleatum* infection was also found to promote the proliferative ability of oral carcinoma cells by causing DNA damage through the Ku70/p53 pathway [78].

## Conclusion

**Figure 3:**
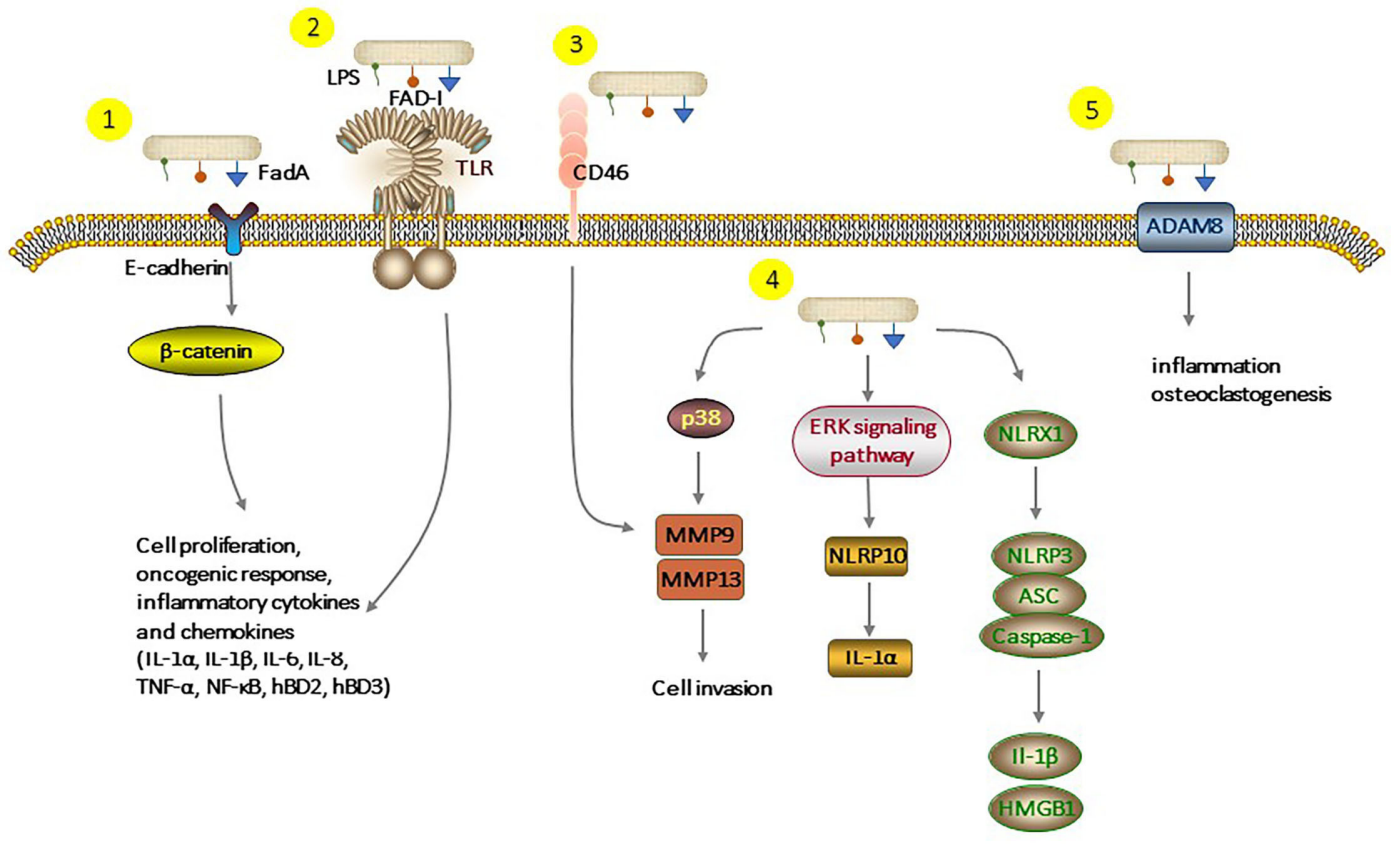
Different pathways of interaction of *F. nucleatum* with epithelial cells. (1), FadA binding to E-cadherin activates β-catenin signaling, leads to cell proliferation, oncogenic, and inflammatory responses. (2), FAD-I can induce hBD2 expression *via* both TLR-1/2 and TLR-2/6. LPS and cell extracts of *F. nucleatum* can also increase production of inflammatory cytokines and chemokines. (3), *F. nucleatum* binds to CD46 which results in the overexpression of MMP-9. (4), *F. nucleatum* triggers the expression of MMP-9 and MMP-13 over the p38 signaling pathway, which causes invasion into the epithelial cell. Activation of the extraregular regulated protein kinases (ERK) signaling pathway augments the pro-inflammatory cytokine IL-1α levels. *F. nucleatum* infection-triggered inflammatory response activates the NLRP3 inflammasome, which is enhanced by NLRX1. (5), Stimulation by *F. nucleatum* leads to upregulation of ADAM8 expression which is involved in inflammation and essential for osteoclastogenesis. LPS, Lipopolysaccharides; Fad-I, *Fusobacterium*-associated defensing inducer; FadA, Fusobacterial adhesion; NLRP, Nucleotide-binding oligomerization domain-like repeat protein; NLRX, Nucleotide-binding domain and leucine-rich-repeat-containing family member X; ADAM8, A disintegrin and metalloproteinase 8; ASC, Apoptosis-associated speck-like protein containing a carboxy-terminal CARD; MMPs, Matrix metalloproteinase.

*Fusobacterium nucleatum*, as one of the resident members of the oral microflora, plays a significant role in the pathogenesis and progression of periodontitis. Long-term infection with *F. nucleatum* may lead to the development of cancer. Several studies demonstrated that *F. nucleatum* could be a causative constituent of oral squamous cell carcinoma. There are some underlying molecular mechanisms about *F. nucleatum* and oral epithelial cells, but we tried to review almost all known mechanisms, which are summarized in Figure 3. *F. nucleatum* adheres to and invades oral epithelial cells by binding host receptors, modulating host signaling pathway and cytokine network. This induced an inflammatory environment in the host and promoted the secretion of inflammatory factors and serine proteases, disrupting the immune-inflammatory balance and damaging periodontal tissue. Moreover, chronic infections are related to the increased risk of cancer. The interaction between *F. nucleatum* and oral epithelial cells creates a microenvironment that promotes tumor growth. Although growing evidence has contributed to a better understanding of the *F. nucleatum*-GEC interactions and how this dialog modulates host responses leading to inflammatory diseases, future studies are needed to identify new potential molecular targets for preventing and treating *F. nucleatum*-induced diseases.

## Author Contributions

SG wrote the manuscript together with YZ. SR and JM helped to prepare the manuscript. All authors contributed to the article and approved the submitted version.

## Conflict of Interest

The authors declare that the research was conducted in the absence of any commercial or financial relationships that could be construed as a potential conflict of interest.

## Publisher's Note

All claims expressed in this article are solely those of the authors and do not necessarily represent those of their affiliated organizations, or those of the publisher, the editors and the reviewers. Any product that may be evaluated in this article, or claim that may be made by its manufacturer, is not guaranteed or endorsed by the publisher.
